# Investigating upper urinary tract urothelial carcinomas: a single-centre 10-year experience

**DOI:** 10.1007/s00345-016-1820-8

**Published:** 2016-04-29

**Authors:** Harveer S. Dev, Stephanie Poo, James Armitage, Oliver Wiseman, Nimish Shah, Samih Al-Hayek

**Affiliations:** Department of Urology, Cambridge University Hospital NHS Trust, Addenbrookes Hospital, Cambridge, CB20QQ UK

**Keywords:** Upper urinary tract urothelial carcinoma, Ureteroscopy, Cytology, Endoscopic biopsy, Tumour grade

## Abstract

**Objectives:**

Evidence of the accuracy of predictive tests in confirming the presence and grade of upper urinary tract urothelial carcinomas (UUTUC) is limited. We present the largest series evaluating the diagnostic value of pre- and intra-operative parameters in the detection of UUTUC.

**Materials and methods:**

We retrospectively analysed records of patients who underwent diagnostic ureteroscopy between 2005 and 2014 for suspected UUTUC. Pre-operative workup included voided urine cytology and CT imaging. Intra-operative assessments involved ureteroscopy to directly visualise suspicious lesions, and where possible selective cytology and biopsy. Primary outcomes were the visualisation of UUTUC and histopathological confirmation of tumour.

**Results:**

Hundred out of 160 (63 %) patients presenting with suspected upper tract malignancy had UUTUC. Voided and selective urine cytology and CT individually predicted UUTUC with a sensitivity/specificity of 63/67, 76/73, and 95/26 %, respectively. Forty out of 48 (83 %) patients who had abnormal CT and abnormal voided urine cytology had UUTUC, while 100 % of those with normal CT and normal voided cytology (investigated for ongoing symptoms) were normal. Comparing endoscopic biopsy to nephroureterectomy specimen grade, 19 (46 %), 18 (44 %), and 4 (10 %) were identical, upgraded, and downgraded, respectively.

**Conclusion:**

Pre-operative investigations can predict UUTUCs. When these investigations were normal, the risk of UUTUC is negligible. In selective patients with abnormal investigations, ureteroscopy should be performed to confirm and predict the grade of UUTUC, in order to guide future management. Selective cytology is unlikely to significantly contribute to the diagnostic workup of UUTUC.

**Electronic supplementary material:**

The online version of this article (doi:10.1007/s00345-016-1820-8) contains supplementary material, which is available to authorized users.

## Introduction

Upper urinary tract urothelial carcinoma (UUTUC) is a rare malignancy, accounting for 5 % of all urothelial cancers [11]. Few useful prognostic factors have been established, and the 5-year specific survival remains low, at <50 % for pT2 and pT3 diseases, and <10 % for pT4 disease [6].

Radical nephroureterectomy (RNU) with excision of a bladder cuff remains the gold standard treatment for UUTUC, regardless of the location of the tumour in the upper urinary tract [9]; however, improvements in endoscopic techniques have led to increasing number of patients being managed endoscopically.

The natural history of UUTUC is still poorly understood, and accurate risk stratification remains elusive. Attempts have been made to incorporate clinical and pre-operative parameters into risk stratification tools [3, 13], which included urine cytology and imaging studies such as multidetector computed tomography urography (MDCTU or CT) [4]. This is in addition to ureteroscopic findings, which have shown to be important in identifying and predicting the progression of UUTUC [2]. Early diagnosis is crucial to tailor individualised management plans and improve outcomes, and delays between diagnosis and surgery have shown to have negative implications on disease recurrence and cancer-specific mortality [8].

Our study evaluates the diagnostic and prognostic value of the pre-operative and intra-operative parameters utilised in the investigation of UUTUC.

## Patients and methods

### Study population

**Fig. 1:**
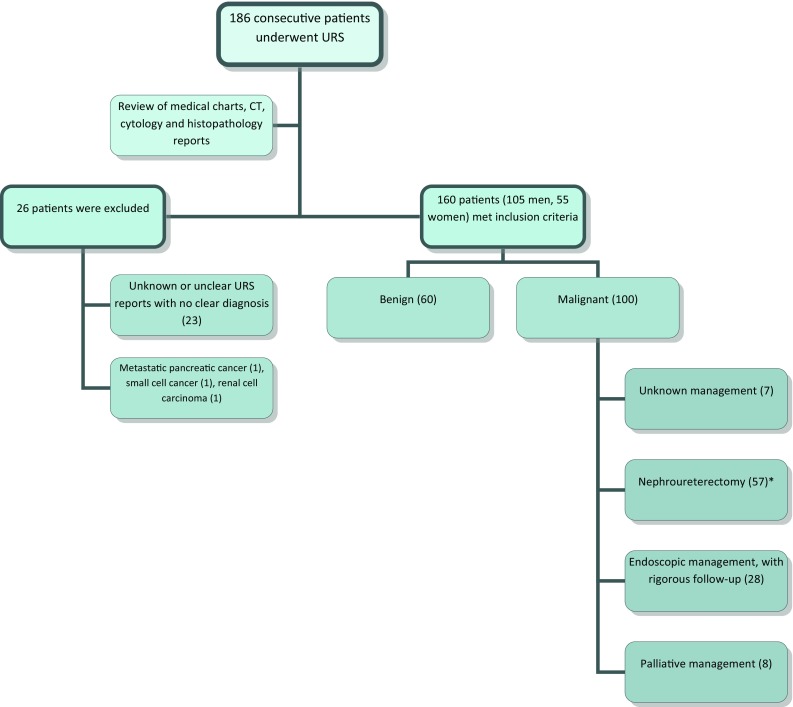
Patient selection criteria. *2 of which were later confirmed to be benign on final histopathological analysis of nephroureterectomy specimens

We retrospectively analysed consecutive patients who underwent ureteroscopy (URS) at our centre between January 2005 and March 2014. Patients were referred to our urology service with a suspicion of malignancy based on urinary tract symptoms (including haematuria, loin pain, and recurrent urinary tract infections). Following initial evaluation with flexible cystoscopy and upper tract imaging, patients were counselled and offered further upper tract assessment with rigid and/or flexible URS if they had abnormal cytology, abnormal CT, ongoing haematuria, or abnormality from ureteric orifice at flexible cystoscopy. The diagnosis of UUTUC was based on the presence of distinct papillary or solid tumours observed on URS, and where available, histopathology results from ureteroscopic biopsies and nephroureterectomy specimens. The medical charts, radiological, cytological, and pathological reports for all patients were independently reviewed by two authors (HD and SP). The clinicopathological parameters collected were gender; age and clinical presentations at diagnosis; previous history of bladder cancer; and patient treatment (endoscopic, radical surgery, or palliation). Twenty-six patients were excluded due to other malignancies or incomplete information (Fig. 1).

### Pre-operative investigations

Both pre-operative ‘voided’ and intra-operative ‘selective’ cytology reports were assessed. Selective cytology was obtained during ureteroscopic washings of the renal pelvis and/or ureters. Cytology samples were classified as negative, atypical, and positive. Positive cytology was used when a high proportion of cells carried a high index of suspicion for malignant UUTUC, possessing exceedingly abnormal morphology. Atypical cytology was defined by the presence of a few abnormal cells, insufficient to exclude malignancy. CT reports were obtained prior to URS, and three main outcomes were evaluated: the presence or absence of (1) a filling defect or a soft tissue mass; (2) hydronephrosis/hydroureter; and (3) thickening of the ureteric wall. As such, an abnormal CT was defined as one which demonstrated a filling defect in the excretory phase, a visible mass in the region of the pelvis or ureter; hydroureter/hydronephrosis or a ureteric stricture; a thickening of the ureteric wall with or without peri-ureteric stranding.

For each of these variables, the sensitivities and specificities were calculated, with respect to the presence of UUTUC, and for nephroureterectomy specimens with respect to tumour invasiveness.

### Histopathological assessment

All biopsies and histopathological specimens were reviewed by our institution’s histopathologists, based on 2004 World Health Organisation’s (WHO) and International Society of Uropathologists (ISUP) grading system [5, 7]. PUNLMP grades 1 and 2 were defined as low-grade, while grade 3 was classified as high-grade disease. Pathology stage was used as a marker of tumour invasiveness [12], with invasive tumours defined as pT1 and above.

### Statistical analysis

We performed Pearson’s Chi-squared tests to evaluate the relationship between pre-operative variables and intra-operative variables, and calculated the sensitivities and specificities. We also conducted two separate multivariable regression analyses and calculated the corresponding area under the curve. All results were two-sided and deemed statistically significant if the *p* value <0.05. Statistical analyses were performed using IBM SPSS Statistics for Windows^®^, version 22.0. Armonk, NY: IBM Corp.

## Results

### Patients

Out of 186 patients who underwent diagnostic URS, 160 patients were included in the analysis (Fig. 1). The patient characteristics, demographics, and presenting symptoms are outlined in Table 1. Patients underwent diagnostic URS (rigid ± flexible) if they had abnormal cytology (*n* = 59, 51 %), abnormal CT (*n* = 135, 85 %), ongoing haematuria (*n* = 3, 2 %), or abnormality from ureteric orifice at flexible cystoscopy (*n* = 2, 1 %). The proportion of patients who underwent each diagnostic investigation is shown in Supplemental Figure 1.

**Table 1:** Patient demographics and characteristics

Variable	Benign (%)	Malignant (%)	Total (%)
Total	60 (100)	100 (100)	160 (100)
Male	33 (55)	72 (72)*	105 (66)
Female	27 (45)*	28 (28)	55 (34)
Median age at presentation (IQR)	66 (50–76)	73 (66–81)	71 (61–78)
Presenting symptoms			
Haematuria	36 (60)	56 (56)	92 (58)
UTI/loin pain	14 (23)	12 (12)	26 (16)
Associated history of bladder TCC	17 (28)	45 (45)*	62 (39)
Affected side			
Right	20 (33)	38 (37)	58 (36)
Left	27 (45)	55 (55)	82 (51)
Bilateral	13 (22)	7 (7)	20 (13)

(*) Denotes statistical significance (*p* < 0.05)

Hundred (63 %) patients had UUTUC confirmed at URS and/or after nephroureterectomy (Fig. 1). Malignancy was defined by the presence of UUTUC when directly visualised, and where available, from biopsies taken endoscopically and/or confirmed after nephroureterectomy.

There was a general trend towards differing rates of previous bladder TCC between the benign cohort and those found to have an UUTUC (*χ* ^2^ = 10.53, *p* = 0.062). Thirty-three and 18 % of patients with or without an UUTUC had a prior history of CIS, grade 2 or grade 3 TCC of the bladder (see Supplemental Table 1).

### Pre-operative investigations

**Fig. 2:**
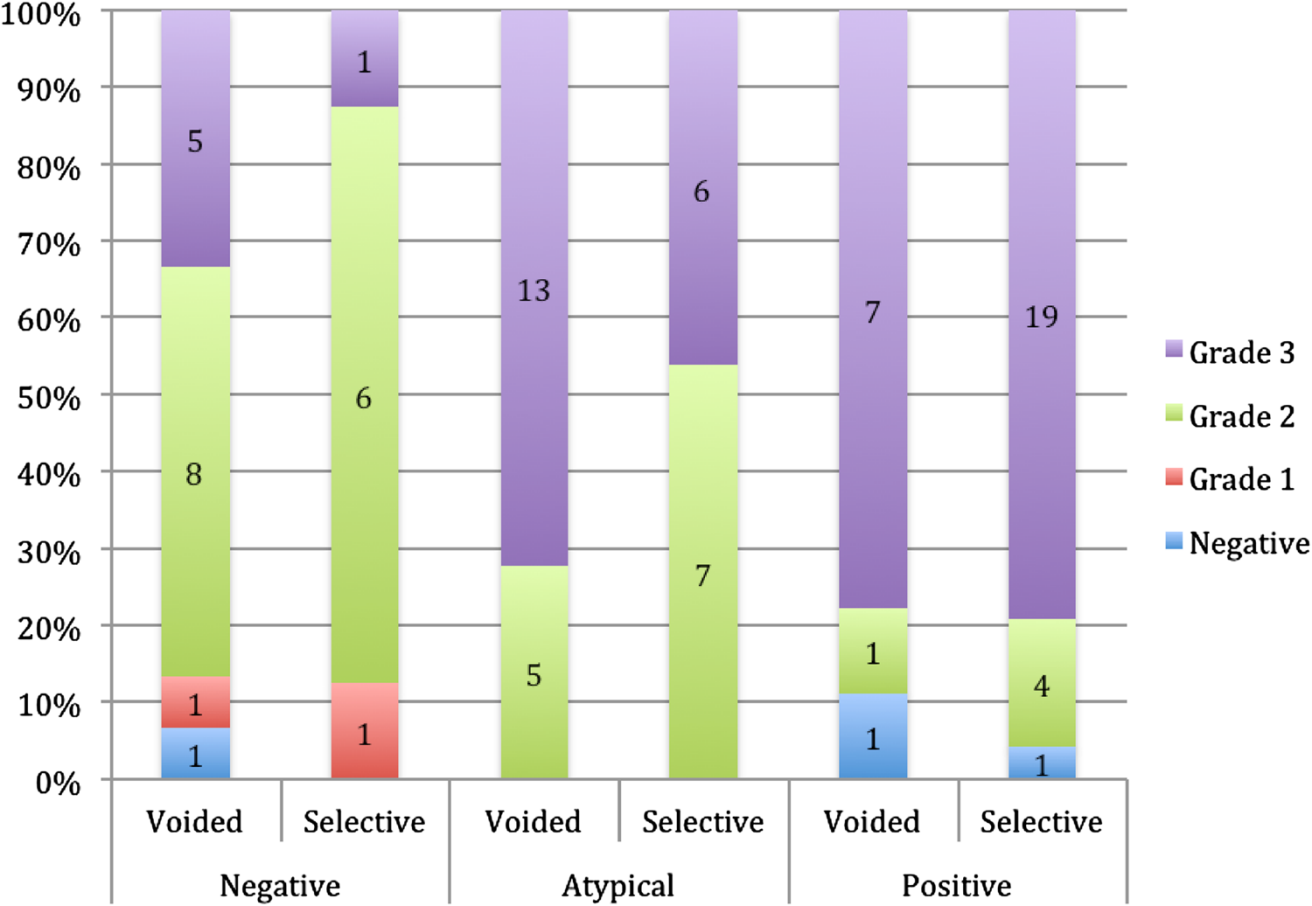
Distribution of voided and selective urine cytology results according to nephroureterectomy specimen grade

Voided urine cytology was obtained in 116 (73 %) patients, of which 57 (49 %) were negative, 31 (27 %) were atypical, and 28 (24 %) were positive (Supplemental Figure 2a). Abnormal (i.e. atypical or positive) voided urine cytology predicted the presence of UUTUC (*χ* ^2^ = 10.165, *p* = 0.006) with a sensitivity and specificity of 63 and 67 %, respectively, and predicted tumour invasiveness (*χ* ^2^ = 6.67, *p* = 0.048). Figure 2 shows the proportions of each voided cytology parameter relative to the final histopathological grade. Voided urine cytology was able to predict G2 and G3 diseases on final histopathological specimens at 43 and 80 % sensitivities, respectively. In addition, voided urine cytology was also able to significantly predict selective cytology obtained endoscopically (Supplemental Figure 2c), with only 10 extra malignancies being identified by selective ureteroscopic washings. Of note, all of these ten additional patients also had abnormalities on CT (eight with filling defects or mass, one with ureteric thickening and one with hydronephrosis/hydroureter).

All 160 patients who underwent a URS had a CT, which identified a filling defect or mass in 100 (63 %) cases. The proportions of each parameter identified on CT imaging and their respective ability to predict UUTUC are summarised in Table 2. Abnormal CT predicted UUTUC with a sensitivity of 95 % and specificity of 26 % (Table 2); however, CT findings were unable to predict tumour invasiveness (data not shown). Of the three CT parameters (filling defect or mass, ureteric thickening, or hydronephrosis/hydroureter) evaluated individually, a filling defect or soft tissue mass detected on CT was the only significant predictor of malignant status (*χ* ^2^ = 15.213, *p* < 0.001). Five (25 %) patients with normal CTs had UUTUC identified on URS, while 15 (75 %) with normal CTs were correctly confirmed after URS; 42 (31 %) patients with abnormalities on CT had a normal URS.

**Table 2:** CT findings and prediction of UUTUC

CT	Malignant status (%)		
	Benign	Malignant	Total
Normal	15 (75)*	5 (25)*	20
Abnormal	42 (31)*	93 (69)*	135
Hydronephrosis/hydroureter	16 (29)	39 (71)	55
Ureteric thickening	12 (35)	22 (65)	34
Filling defect or soft tissue mass	28 (28)*	72 (72)*	100
Total			155

Single findings on CT urogram	Benign	Malignant	Total
Hydronephrosis	8 (47)	9 (53)	17
Thickening	5 (56)	4 (44)	9
Filling defect or soft tissue mass	12 (24)*	37 (76)*	49

Malignant status is determined by the presence of UUTUC observed on URS and/or histologically defined from biopsies taken endoscopically or following nephroureterectomy. CT reports were available in 155 out of 160 patients. The term ‘abnormal’ refers to the detection of filling defect or soft tissue mass, hydronephrosis/hydroureter or ureteric thickening on CT. Percentages of row totals are denoted in brackets. (*) denotes a statistically significant difference between columns

UUTUC was detected in 33/39 (85 %) patients who had filling defects or mass reported on CT together with abnormal voided urine cytology (17/20 with positive cytology, and 16/19 with atypical cytology) (Table 3). Similarly, 47/55 (85 %) patients were identified with UUTUC having had filling defects or mass on CT and abnormal selective cytology.

**Table 3:** Correlations between CT imaging and cytological findings

CT	Voided urine cytology (%)				Selective urine cytology (%)			
	Negative		Atypical or positive		Negative		Atypical or positive	
	Benign	Malignant	Benign	Malignant	Benign	Malignant	Benign	Malignant
Normal	5 (100)	0 (0)	5 (71)	2 (29)	10 (100)	0 (0)	4 (80)	1 (20)
Relevant Pathology	9 (56)	7 (44)	2 (22)	7 (78)	6 (50)	6 (50)	2 (20)	8 (80)
Filling defect/mass	19 (53)	17 (47)	6 (15)	33 (85)	19 (61)	12 (39)	8 (15)	47 (85)

Eight (17 %) patients with positive voided cytology and abnormality on CT demonstrated a benign histology; six (75 %) of these had an identifiable filling defect or mass on CT.

### Selective cytology

Selective cytology was obtained during URS in 126 (79 %) patients, where 55 (44 %), 27 (21 %), and 44 (35 %) were reported as negative, atypical, and positive, respectively. Selective cytology predicted UUTUC (*χ* ^2^ = 30.866, *p* < 0.001) with a sensitivity and specificity of 76 and 73 %, respectively, in addition to predicting tumour invasiveness (*χ* ^2^ = 8.608, *p* = 0.197) (Supplemental Figure 2b). Figure 2 shows the proportions of each selective cytology outcome relative to the final grade from nephroureterectomy specimens. The prognostic ability of selective cytology was only marginally better than pre-operative voided cytology. In G2 and G3 nephroureterectomy specimens, the respective sensitivities for selective cytology was 65 and 96 % compared with 43 and 80 % for voided urine cytology (data not shown).

Of those with available selective cytology results and subtle CT findings (hydronephrosis/hydroureter or ureteric thickening only) (Table 2), eight had a benign outcome (with two abnormal and six negative selective cytology results); eight had a malignant outcome (four abnormal and four negative selective cytology results).

### Ureteroscopic biopsy

Table 4 shows the histopathology from ureteroscopic biopsies and post-nephroureterectomy specimens, stratified according to grade and stage. Of 75 ureteroscopic biopsies that were taken, 65 (87 %) had UUTUC present. Of 59 gradable specimens, 38 (64 %) were low-grade, while 21 (36 %) were high-grade disease; 34 (64 %) tumours were invasive, and 19 (36 %) were non-invasive. Fifty-five out of 57 (96 %) nephroureterectomy specimens were identified as malignant; 2 (3 %) final cases had previously identified malignancy on URS, where the tumour was likely sufficiently removed by biopsy or diathermy rendering the final post-nephroureterectomy histopathological diagnosis as benign.

**Table 4:** Distribution of ureteroscopic biopsy and post-nephroureterectomy specimen histopathology, stratified according to tumour grade and stage

Variable	Ureteroscopic biopsies (%)	Nephroureterectomy specimens (%)
Total	75	57
Negative	10 (12)	2 (4)
UUTUC	65 (87)	55 (96)
Grade		
Total	75	57
Negative	11 (15)	2 (4)
UUTUC NOS^a^	5 (7)	4 (7)
G1	8 (11)	1 (2)
G2	30 (40)	19 (33)
G3	21 (28)	31 (54)
Stage		
Total	67	55
Negative	11 (16)	–
UUTUC NOS^a^	2 (3)	2 (4)
pTa	45 (67)	15 (27)
pTis	–	4 (7)
pT1	6 (9)	9 (16)
pT2	3 (4)	5 (9)
pT3	–	17 (31)
pT4	–	3 (6)

^a^UUTUC NOS denotes malignant UUTUC not otherwise specified

**Fig. 3:**
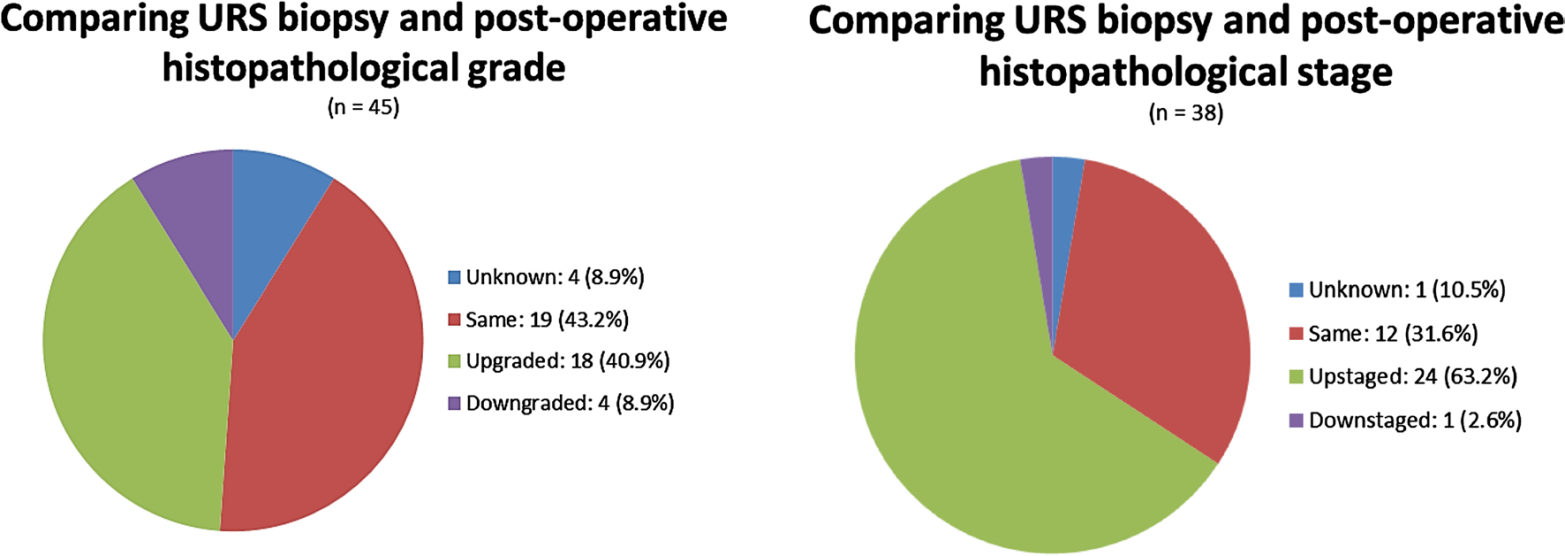
Comparison of ureteroscopic biopsy and post-operative nephroureterectomy specimen grade (*left*) and stage (*right*)

Ureteroscopic biopsy positively correlated with nephroureterectomy specimen in terms of grade (*χ* ^2^ = 19.793, *p* = 0.071) and stage (*χ* ^2^ = 19.950, *p* = 0.336) (Fig. 3). Nineteen (43 %) and 12 (32 %) biopsies matched the grade and stage of nephroureterectomy specimens. As expected, the proportion of biopsies that were upgraded and up-staged was higher than those downgraded and down-staged (Fig. 3). Seven cases of 41 cases which could be analysed were upgraded to G2 or G3 disease (data not shown).

### Multivariable analysis of pre-operative and intra-operative variables

Pre-operatively, male gender, age, voided urine cytology, and the presence of filling defect or mass on CT imaging were shown to be robust independent predictive factors of UUTUC, with the latter demonstrating the highest hazard ratios of 11.7 (Table 5); AUC 0.816 (95 % CI 0.73–0.90). Ureteric thickening and hydronephrosis/hydroureter on CT were not significant independent predictors of UUTUC.

**Table 5:** Multivariable regression analysis of pre-operative variables predicting the presence of UUTUC

Variable	Exp(*B*)	95 % CI	*p* value
*Pre-operative factors*			
Male versus female (ref)	3.025	1.080–8.474	0.035
Age	1.060	1.019–1.102	0.004
Pre-operative urine cytology			
Negative (ref)			0.018
Atypical	4.980	1.518–16.331	0.008
Positive	2.557	0.843–10.663	0.090
*CT*			
Normal (ref)			0.014
Hydronephrosis/hydroureter or ureteric thickening	6.643	0.890–49.571	0.065
Filling defect/soft tissue mass	11.675	1.819–71.817	0.009

## Discussion

Our results confirm that, pre-operatively, voided urine cytology and the presence of filling defect or mass on CT are important positive findings in the investigation of suspected UUTUC. In addition to gender and age, they were able to independently predict the presence of UUTUC.

There were high false-positive rates particularly from CT investigations and non-specific findings of hydronephrosis/hydroureter and ureteric thickening. Specifically, these findings on CT were not able to significantly predict the presence of tumour compared to the conclusions of another study [1]. The combined sensitivity of voided urine cytology and CT of 85 % demands the use of URS to correctly identify patients with UUTUC. In contrast, since 100 % of those with both a normal CT and normal voided cytology had benign findings on URS (Table 3), we would propose that with clinical judgment, such patients, individually risk-stratified, would not require URS. Clearly, equivocal results in either modality may necessitate further investigation.

A limited analysis of 6/39 (15 %) benign cases after URS, who had demonstrated measurable mass or filling defects on CT, was undertaken. While the presence of a measurable mass is more indicative of malignancy (approximately 2:1 incidence rates in malignant and benign pathology, respectively—data not shown), we were unable to identify a cut-off size of a mass on CT that would be able to discern between benign and malignant findings.

As expected, voided urine cytology correlated well with selective cytology, although multivariate analysis suggests that selective cytology has statistically superior predictive capability. However, in terms of clinical utility, when directly compared to selective cytology, this only identified 10 additional malignant cases over voided cytology. We found 85 % of patients with both abnormal CT and abnormal voided cytology had an UUTUC. The same proportion (85 %) was identified in those with abnormal CT and abnormal selective cytology. Indeed, our results suggest that the addition of selective cytology provides only marginal additional value. Furthermore, the combination of an abnormal selective cytology with subtle CT findings (thickening or hydronephrosis/hydroureter alone) was not sufficient to predict the presence of UUTUC, and direct visual observation and/or confirmation with biopsy was critical in determining clinical need for nephroureterectomy in these cases.

Previous attempts have been made correlating histology of ureteroscopic biopsies with nephroureterectomy specimens, but these have been limited by small case numbers [3]. We present the largest such series and demonstrate that biopsies do correlate with high-grade disease, and relatively few cases require upgrading, which may reflect more systematic technical approaches to the biopsying of tumours.

In the absence of sufficient staging data, we attempted to identify the predictive capacity of a high-grade biopsy to predict UUTUC invasiveness; however, after multivariate analysis, a high-grade biopsy was unable to predict UUTUC invasiveness in our series. Smith et al. [10] suggested that biopsy grade may still be subject to a non-negligible rate of sampling error, particularly with larger tumours, which may in part explain these results. It is possible that employing narrowband imaging technology, better characterised for the diagnosis of bladder TCC, may still further improve the identification and biopsy of suspected UUTUC, and this will be an important variable to consider in future studies as these ureteroscopes become more widely available.

The study is limited by variations between reporting radiologist and histopathologists, although all followed standard protocols employed at our institution and undergo robust quality control measures. Visualisation of lesions was used to define the presence of malignancy in some cases, and although this was restricted to clear papillary lesions, there is an inherent limitation when a biopsy is not taken. The study data are also from a single institution, with retrospective data collection, and lacked a comprehensive account of potential aetiological risk factors for each patient. Future work could benefit from prospective data collection, standardised reporting on URS and pathological specimens, and multi-institutional data collection, to further improve statistical power and clinical applicability.

## Conclusion

Our study represents the largest series in the UK to evaluate and quantify the predictive capabilities of the pre-operative and intra-operative parameters in the diagnosis of UUTUC. We support the strategy whereby voided urine cytology and CT are useful as screening tools to identify at-risk patients. When abnormalities were detected in both pre-operative voided cytology and CT, malignancy was confirmed in 85 % cases. URS is imperative in identifying benign cases, particularly when the CT findings represent the only abnormal pre-operative investigation. In the presence of a normal CT and voided urine cytology, we propose that a clinical judgment can be made for not performing a URS, provided that individual patient risk factors are also taken into account.

Selective urine cytology does not appear to clinically improve the prediction of UUTUC. However, URS and biopsy remain a valuable confirmatory tool for diagnosis, therapy, and guiding surgical management. Our results provide quantitation of the current diagnostic tools utilised in clinical practice, and in combination with multi-institutional datasets could contribute towards more robust tools of risk stratification.

## Electronic supplementary material

Below is the link to the electronic supplementary material.

Supplementary material 1 (DOCX 148 kb)
