# Supplementary material for: Investigating upper urinary tract urothelial carcinomas: a single-centre 10-year experience
Source: World J Urol. 2016 Apr 29;35(1):131–8. doi: 10.1007/s00345-016-1820-8 (PMC5233745; doi:10.1007/s00345-016-1820-8)
Supplement: Supplementary file 1 — Supplementary material 1 (DOCX 148 kb) [file 345_2016_1820_MOESM1_ESM.docx]

**SUPPLEMENTAL FIGURES AND TABLES**

**Supplemental Figure 1: Summary Graph showing the number of patients (%) who had pre-operative and intra-operative investigations**


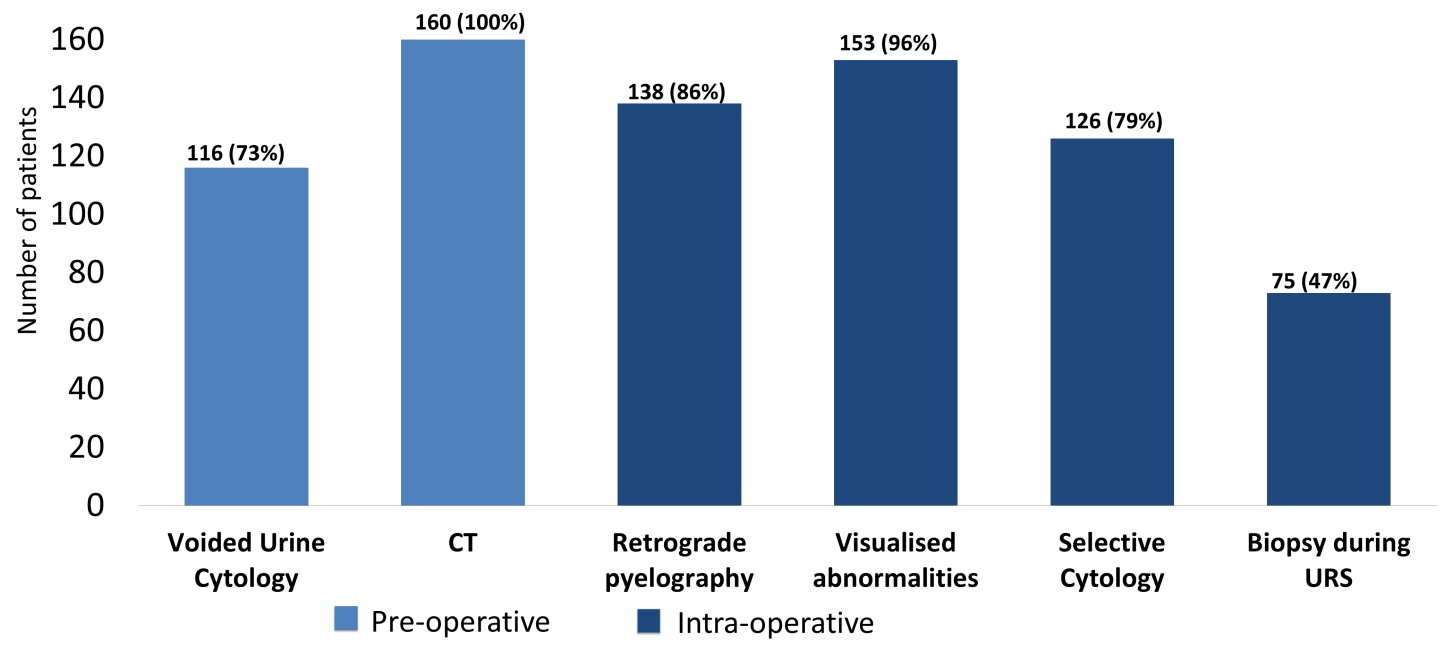


**Supplemental Table 1: Incidence of previous bladder TCC by the presence or absence of UUTUC following investigations.**

|  | | | | | |
| --- | --- | --- | --- | --- | --- |
|  | | | **UUTUC investigation findings** | | Total |
|  |  |  | **Benign** | **Malignant** |  |
| **Bladder TCC** | **No previous Bladder TCC** | Count | 43 | 55 | 98 |
|  |  | % within UUTUC | 71.7% | 55.0% | 61.3% |
|  | **G1** | Count | 4 | 6 | 10 |
|  |  | % within UUTUC | 6.7% | 6.0% | 6.3% |
|  | **G2** | Count | 3 | 15 | 18 |
|  |  | % within UUTUC | 5.0% | 15.0% | 11.3% |
|  | **G3** | Count | 6 | 18 | 24 |
|  |  | % within UUTUC | 10.0% | 18.0% | 15.0% |
|  | **CIS** | Count | 2 | 0 | 2 |
|  |  | % within UUTUC | 3.3% | 0.0% | 1.3% |
|  | **Unknown** | Count | 2 | 6 | 8 |
|  |  | % within UUTUC | 3.3% | 6.0% | 5.0% |
| Total | | Count | 60 | 100 | 160 |
|  |  | % within UUTUC | 100.0% | 100.0% | 100.0% |

**Supplemental Figures 2 a, b, c: Predictive capacity of (2a) voided and (2b) selective cytology to predict the presence of UUTUC or invasive tumour, and; (2c) the correlations between voided and selective urine cytology**

2a

| **Voided urine cytology** | **Malignant status (%)** | | |  | **Tumour Invasiveness (%)** | | | |
| --- | --- | --- | --- | --- | --- | --- | --- | --- |
|  | Benign | Malignant | Total |  | Benign | Non-invasive | Invasive | Total |
| **Negative** | *32 (56)* | *25 (44)* | **57** |  | *1 (7)* | *8 (53)* | *6 (40)* | **15** |
| **Atypical** | *9 (29)* | *22 (71)* | **31** |  | *0* | *2 (12)* | *15 (88)* | **17** |
| **Positive** | *7 (25)* | *21 (75)* | **28** |  | *1 (14)* | *1 (14)* | *5 (72)* | **7** |
| Total |  |  | **116** |  |  |  |  | **39** |

2b

| **Selective urine cytology** | **Malignant status (%)** | | |  | **Tumour Invasiveness (%)** | | | |
| --- | --- | --- | --- | --- | --- | --- | --- | --- |
|  | Benign | Malignant | Total |  | Benign | Non-invasive | Invasive | Total |
| **Negative** | *37 (67)* | *18 (33)* | **55** |  | *0* | *5 (63)* | *3 (17)* | **8** |
| **Atypical** | *8 (30)* | *19 (70)* | **27** |  | *0* | *2 (15)* | *11 (85)* | **13** |
| **Positive** | *6 (13)* | *39 (87)* | **44** |  | *1 (5)* | *5 (23)* | *16 (73)* | **22** |
| Total |  |  | **126** |  |  |  |  | **43** |

2c

| **Cytologic Parameters**  **Voided urine cytology** | **Selective ureteroscopic cytology (%)** | | | | |
| --- | --- | --- | --- | --- | --- |
|  | Negative | | | Atypical or Positive | |
|  | **Benign** |  | **Malignant** | **Benign** | **Malignant** |
| Negative | 23 (72) |  | 9 (18) | 3 (23) | 10 (77) |
| Atypical or Positive | 10 (83) |  | 2 (17) | 5 (14) | 31 (86) |
